# GLAMOUR: GLobAl building MOrphology dataset for URban hydroclimate modelling

**DOI:** 10.1038/s41597-024-03446-2

**Published:** 2024-06-12

**Authors:** Ruidong Li, Ting Sun, Saman Ghaffarian, Michel Tsamados, Guangheng Ni

**Affiliations:** 1 Department of Hydraulic Engineering, Tsinghua Univeristy, Beijing, China; 2 Institute for Risk and Disaster Reduction, University College London, London, UK; 3 Department of Earth Sciences, University College London, London, UK

**Keywords:** Atmospheric science, Hydrology

## Abstract

Understanding building morphology is crucial for accurately simulating interactions between urban structures and hydroclimate dynamics. Despite significant efforts to generate detailed global building morphology datasets, there is a lack of practical solutions using publicly accessible resources. In this work, we present GLAMOUR, a dataset derived from open-source Sentinel imagery that captures the average building height and footprint at a resolution of 0.0009^°^ across urbanized areas worldwide. Validated in 18 cities, GLAMOUR exhibits superior accuracy with median root mean square errors of 7.5 m and 0.14 for building height and footprint estimations, indicating better overall performance against existing published datasets. The GLAMOUR dataset provides essential morphological information of 3D building structures and can be integrated with other datasets and tools for a wide range of applications including 3D building model generation and urban morphometric parameter derivation. These extended applications enable refined hydroclimate simulation and hazard assessment on a broader scale and offer valuable insights for researchers and policymakers in building sustainable and resilient urban environments prepared for future climate adaptation.

## Background & Summary

As our planet grapples with increasing unprecedented hydroclimate hazards induced by climate change, it is essential to understand the spatiotemporal intertwining between intensifying extreme events and evolving human settlements, especially in urbanized area^1^. Buildings, a ubiquitous form of infrastructure in cities, exhibit morphological characteristics that are crucial for devising effective climate-responsive strategies, including future-oriented hydrometeorological simulations^2,3^, disaster risk assessments^4^, and the planning of sustainable cities^5^. Numerous studies have aimed to quantify the horizontal spread of urban areas and track changes in human settlement boundaries globally over decades^6^.

However, refined representation of the complex urban environment also necessitates the incorporation of detailed information about the vertical dimension of buildings^5^. Thus, there have been increasing efforts concerning creating large-scale datasets of 3D building morphology. Biljecki *et al*.^7^ designed a comprehensive list of building-related morphological indicators and implemented a corresponding open-source computational solution based on spatially enabled PostgreSQL database composed of OpenStreetMap buildings (OSM). However, a recent investigation^5^ reveals that for 69.5% of urban agglomerations worldwide, the completeness of OSM data remains below 20%, thus limiting its global applicability. Thanks to the emergence of publicly available and globally distributed satellite imagery, various research has focused on large-scale 3D building structure mapping based on remote sensing based data sources. One straightforward approach is to derive building height as the normalized digital surface model (nDSM) defined by the difference between the corresponding digital terrain model (DTM) and the digital surface model (DSM)^8^. Since most global digital elevation models (DEMs) fall towards the DSM side which detects the elevation of the surface canopy composed of vegetation and man-made structures^9^, the key challenge here is to produce an accurate representation of the terrain ground. While some algorithms have been developed to discern the top and bottom sections of buildings through morphological operations on global DSM datasets (e.g., ALOS AW3D30^10,11^), the limited spatial resolution of publicly accessible topographical data often conflates building height with ground elevation in its measurements and thus introduces significant uncertainty when attempting to deduce building heights from these amalgamated figures using straightforward mathematical transformations. Esch *et al*.^12^ improved nDSM-based approaches by local height variation analysis aiming to find vertical edges in 12 m TanDEM-X DEM as building outlines and finally generated the World Settlement Footprint 3D (WSF3D), which is the first globally consistent three-dimensional building morphology dataset. However, even by 12 m pixel spacing, WSF3D is still prone to produce smoothed height edges and therefore requires empirical post-processing using preassigned correction factors to mitigate the underestimation issues in the original building height values.

Considering potential bottlenecks in directly mapping from medium-resolution topographic data, various studies have proposed machine-learning-based (ML) approaches to establish a statistical regression relationship between multi-source data and the 3D structure of buildings. Li *et al*.^13^ fused optical, Synthetic Aperture Radar (SAR) images and corresponding derived indices by the Random Forest (RF) model and generated continental-scale 3D building structures for Europe, the United States and China at 1000 m resolution. Considering that training ML models requires numerous reference samples, Ma *et al*.^14^ proposed to improve their spatiotemporal consistency and retrieval efficiency with GEDI-derived relative height samples using large-scale spaceborne lidar measurement and produced a 150-m building height map in China’s urban agglomerations by the RF model. Regardless of their high interpretability and convenient deployment through cloud platforms like Google Earth Engine (GEE), traditional ML models tend to suffer saturation problems in the high value region^15,16^, which promotes the development of more advanced deep-learning-based (DL) models in 3D building morphology mapping^17–19^. However, a global-scale building morphology dataset at a finer resolution remains absent, lacking both open-source solutions and practical DL-based engineering pipelines.

We introduce GLAMOUR – GLobAl building MOrphology dataset for URban hydroclimate modelling – a comprehensive dataset featuring average building footprint and height data at a resolution of 0.0009^°^ (approximately 100 m at the equator) across 13189 urban areas globally as of 2020. This dataset optimally leverages multi-task DL (MTDL) models, publicly accessible satellite images in conjunction with the Google Cloud ecosystem to enable efficient and accurate large-scale mapping. This up-to-date building morphology dataset provides an unprecedented possibility for enabling various urban hydroclimate applications at a global scale, including human thermal comfort simulation^20^, building energy modelling^21^, 3D flood risk analysis^22^ among others. Additionally, we offer open access to the code for the generation of this dataset through the SHAFTS package^16^, which allows interested users to employ our optimized pipelines to regions of interest (ROI) with the latest released satellite datasets.

## Methods

### Production workflow

In the context of GLAMOUR, following previous research on building morphology mapping, we define the average building footprint *λ*_*p*_ and the area-weighted average building height H_avg_^2,15^ as follows:

1 $${\lambda }_{p}=\frac{\sum {A}_{i}}{{A}_{T}}$$

2 $${H}_{{\rm{avg}}}=\frac{\sum {h}_{i}{A}_{i}}{\sum {A}_{i}}$$

where *A*_*T*_ is the total area of a single grid, *A*_*i*_ is the intersection area of the building *i* with the grid, *h*_*i*_ is the height of building *i*.

**Fig. 1:**
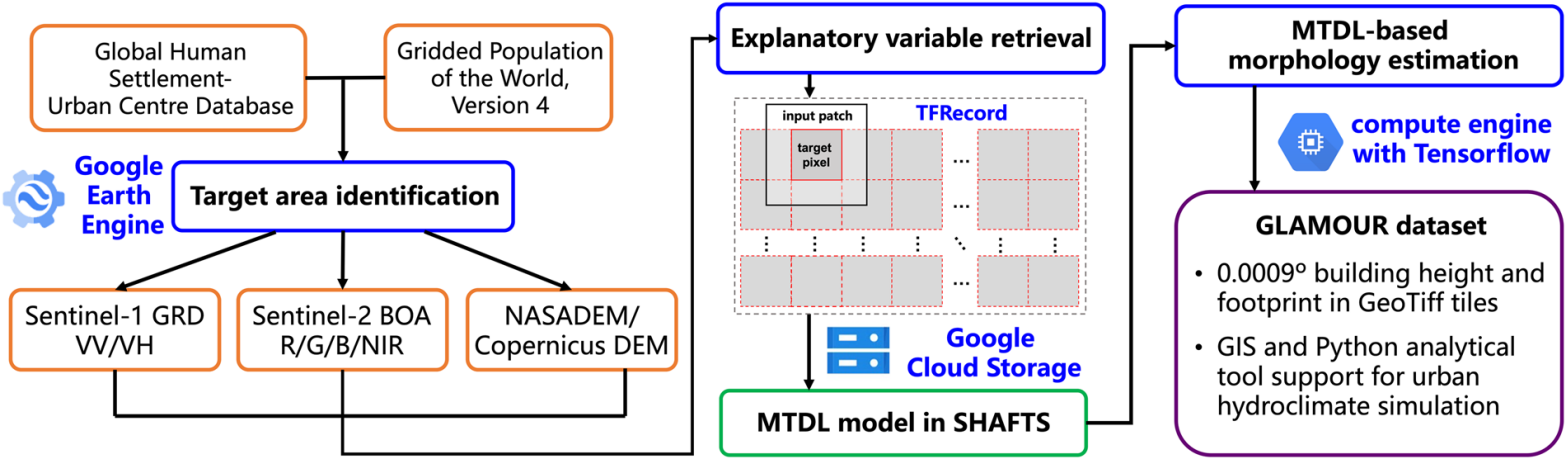
Production workflow of GLAMOUR.

The GLAMOUR production workflow consists of three stages (Fig. 1):

- **Target area identification**: Given the target group being the urban hydroclimate modelling community, the focus of GLAMOUR is set to urbanized areas outlined by the Gridded Population of the World, Version 4 (GPWv4) dataset^23^ and the Global Human Settlement-Urban Centre Database (GHS-UCDB)^24^. Specifically, adhering to the concept of the Degree of Urbanisation approved by the 51st Session of the United Nations Statistical Commission^25^, we first construct spatial grids of 0.09^°^ across the globe and then identify a grid as a potential urbanized area if it satisfies at least one of the following conditions: a) it intersects with 13189 urban centers defined by the GHS-UCDB; or b) its population density exceeds 300 people per km^2^ based on the GPWv4 dataset. We then further smooth the boundary formed by originally identified grids using a morphological closing operation with 3 × 3 rectangular kernels^26^, which finalizes the mapping extent of ROIs considered in the GLAMOUR dataset.
- **Explanatory variable retrieval**: To enable open research, we select publicly accessible satellite images as explanatory variables, including: (1) VV and VH polarizations from Sentinel-1 Ground Range Detected (GRD) data^27^, (2) red, green, blue and the near infra-red (NIR) band from Sentinel-2 Bottom-Of-Atmosphere (BOA) reflectance data^28^, (3) DSM data including NASADEM data for low- and mid-latitude areas(-60.0^°^ < latitude < 60.0^°^)^29^, and Copernicus DEM data for the remaining part^30^ (cf. Table 1). All images have been preprocessed by GEE and can be accessed via its Python interface. We then retrieve them by spatiotemporal filtering and aggregation to minimize the undesirable effects caused by non-man-made elements and cloud blockage (details refer to the later section on processing explanatory variables). After retrieving DSM data and Sentinel-1/2 images from GEE’s image collections, we crop them into 0.0018^°^ × 0.0018^°^ patches centered on each 0.0009^°^ × 0.0009^°^ target pixel. In order to improve the efficiency of subsequent model estimation by batch processing, these 6-band patches are then organized into an array of 100 × 100 covering a geospatial extent of 0.09^°^ × 0.09^°^. Finally, each array is exported as a single TFRecord in the Google Cloud Storage (GCS), ensuring efficient encoding and convenient access for downstream models^31^. To establish a streamlined engineering pipeline for 3D building morphology mapping, we implemented the aforementioned procedures using GEE’s Python interface, which allows for optimized execution in a regular routine.

**Table 1:** Datasets used in the GLAMOUR production.

Data	Type	Resolution	Source
GHS-UCDB data	vector	/	https://ghsl.jrc.ec.europa.eu/ghs_stat_ucdb2015mt_r2019a.php
GPWv4 data	raster	1 km	https://sedac.ciesin.columbia.edu/data/collection/gpw-v4
Sentinel-1 GRD data	raster	10 m	https://documentation.dataspace.copernicus.eu/Data/Sentinel1.html
Sentinel-2 BOA data	raster	10 m	https://documentation.dataspace.copernicus.eu/Data/Sentinel2.html
NASADEM data	raster	30 m	https://cmr.earthdata.nasa.gov/search/concepts/C1546314043-LPDAAC_ECS.html
Copernicus DEM data	raster	30 m	https://spacedata.copernicus.eu/collections/copernicus-digital-elevation-model
- **MTDL-based morphology estimation**: After exporting satellite image patches as TFRecords in GCS, we estimate *λ*_*p*_ and H_avg_ in the target urbanized area by applying an enhanced MTDL model from the SHAFTS package^16^ on multi-band patches (details refer to the later section on the MTDL enhancement). The enhanced MTDL model can achieve simultaneous estimation of building height and footprint using Sentinel imagery and elevation data.

To further refine the representation of urban boundaries in the final product, we perform pixel masking on the initial mapping results by combining the predicted building morphology maps with the World Settlement Footprint layer for 2019 (WSF2019)^6^. To be specific, we exclude a pixel from the original prediction in the GLAMOUR dataset if neither of the following conditions is satisfied:

- predicted *λ*_*p*_ is higher than 0.25.
- more than 20% area is identified as settlement area based on the spatial aggregation results of WSF2019.

After obtaining 3D building morphylogy of 0.09^°^ × 0.09^°^ from each TFRecord, we further mosaick them into larger tiles of 9^°^ × 9^°^ to ensure easy accessibility of the dataset for potential users.

### Processing of explanatory variables

In contrast to static DSM data, Sentinel-1/2 images are regularly acquired by corresponding satellites and thus necessitate appropriate aggregation operations prior to their integration into subsequent estimation models^15^. Sentinel-1 GRD data are collected as SAR measurements and can provide reliable all-weather day-and-night imaging of surface backscattering characteristics influenced by factors including material types and vertical structures^32^.

To mitigate the confounding impact caused by non-man-made elements such as vegetation on building height estimation, we select Sentinel-1 data in the winter season based on the geolocations of ROIs^13^ and aggregate them into the mean value of the corresponding period. For areas uncovered by Sentinel-1 GRD data in winter, we progressively extend the timeframe to include the autumn, spring and summer seasons.

In addition to Sentinel-1 GRD data, we also collect the Sentinel-2 BOA data to capture a more holistic view of the urban landscape through the combination of multi-modal sensors. However, the optical sensors of Sentinel-2 often encounters issues of cloud blockage, which prevent them from clear imaging. To ensure cloud-free Sentinel-2 images for our global mapping tasks, we utilize an aggregation-based engineering approach^33^ to create high-quality mosaics from multi-temporal Sentinel-2 imagery in an automated workflow. To be specific, we filter Sentinel-2 images by the maximum allowable cloud coverage ratio of ROIs or select them based on corresponding quality scores when suitable images are scarce. Once we’ve gathered the desired images, we proceed to mosaic and crop them to fit within the boundaries of the ROI.

### Enhancement to the MTDL model in SHAFTS

Building upon the initial development of SHAFTS, we enlarge our original reference dataset to 116 sample sites including 35 uninhabited sites with zero building footprint and height values which aims to enhance the identification capability of the MTDL model on possible non-man-made vertical objects. Moreover, considering the potential underestimation problem in dense and tall buildings caused by the imbalanced data distribution, we aggregate training samples into specific intervals and then reweight them with the cubic root of their inverse frequency^34^. For sample aggregation, we set the bin width of 5 m and 0.1 for the task of building height and footprint prediction, respectively. Thus, the final training process can be divided into two phases: first, we train all parameters of the MTDL model for 155 epochs using unweighted samples to guarantee the model convergence; then, we finetune the parameters of the last fully-connected layers in the converged MTDL model with weighted samples through an additional 155-epoch training period.

## Data Records

**Fig. 2:**
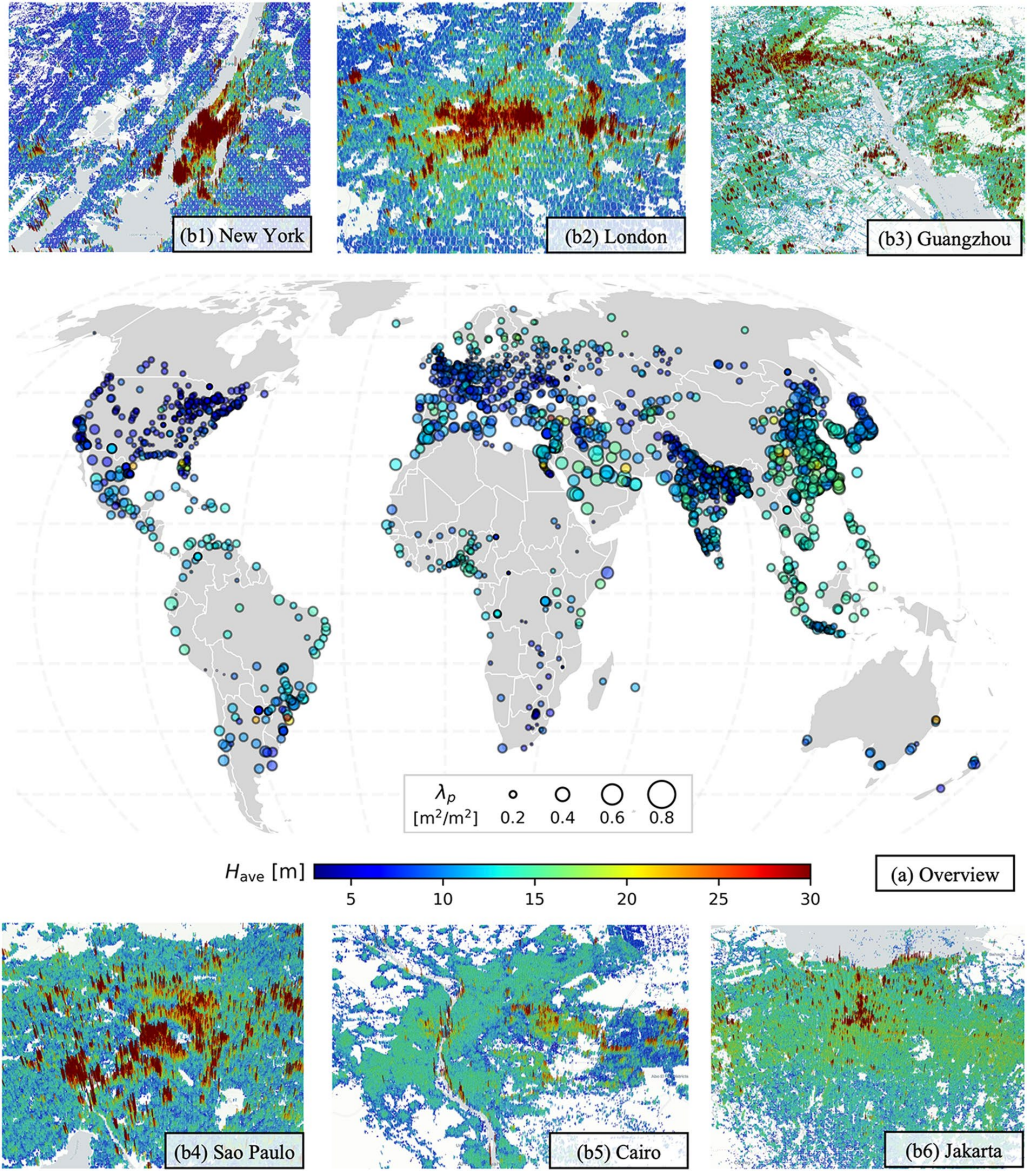
(**a**) Global overview of 3D building morphology in the top 3000 urban centers ranked by area in the GHS-UCDB; (**b**) 3D close-up views for six cities.

GLAMOUR dataset can be accessed at Li *et al*.^35^. This dataset is divided into two subsets for the average building footprint and height at the resolution of 0.0009^°^, respectively. Each subset comprises 261 GeoTiff tiles on 9^°^ grids and can be further visualized and processed in geographic information system (GIS) software. Fig. 2 provides a comprehensive view of the 3D morphological characteristics of buildings resolved by the GLAMOUR dataset in global urban centers defined in the GHS-UCDB as well as several close-up figures to cities located on different continents including New York, London, Guangzhou, Sao Paulo, Cairo and Jakarta.

## Technical Validation

**Fig. 3:**
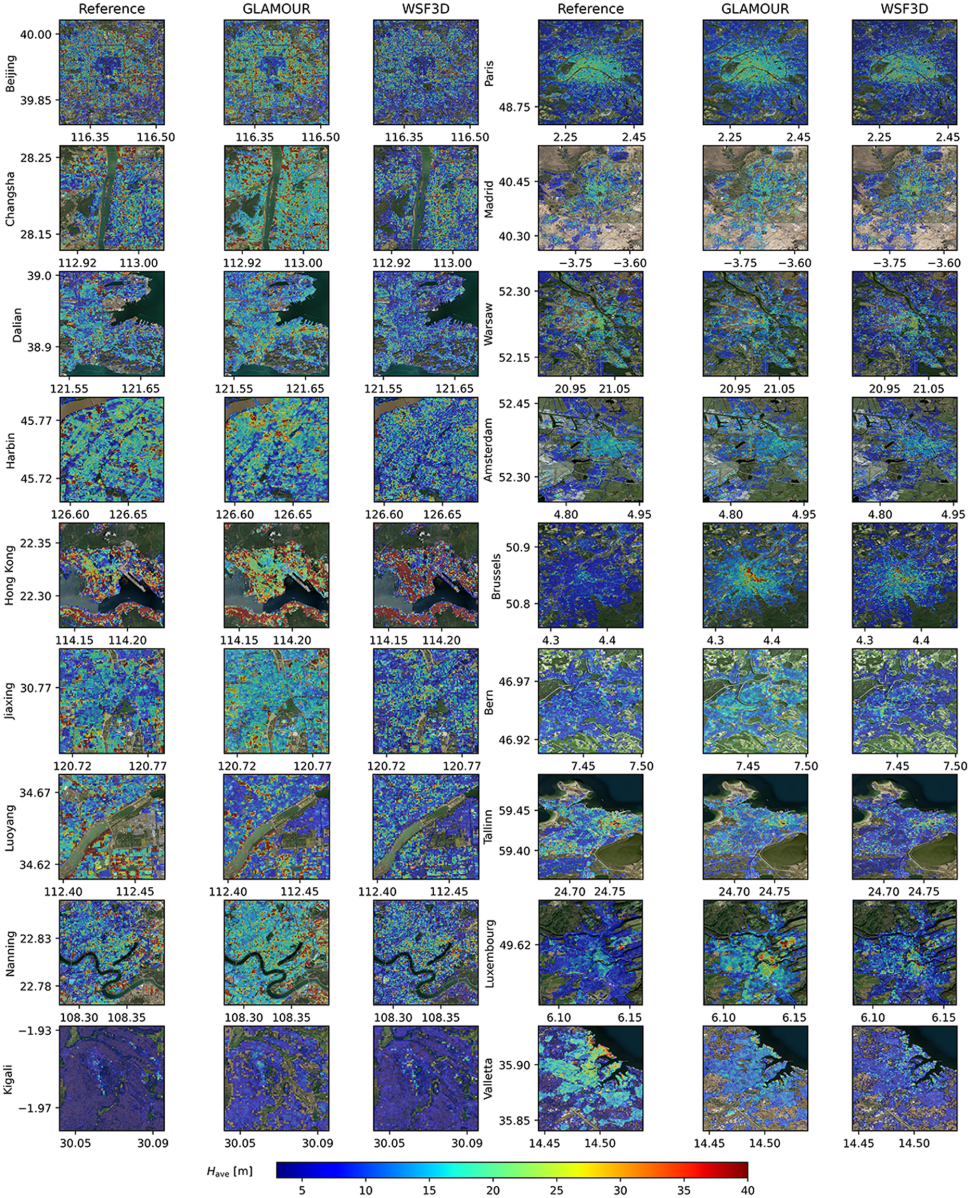
Close-up views of building height (H_avg_) at reference sites.

**Fig. 4:**
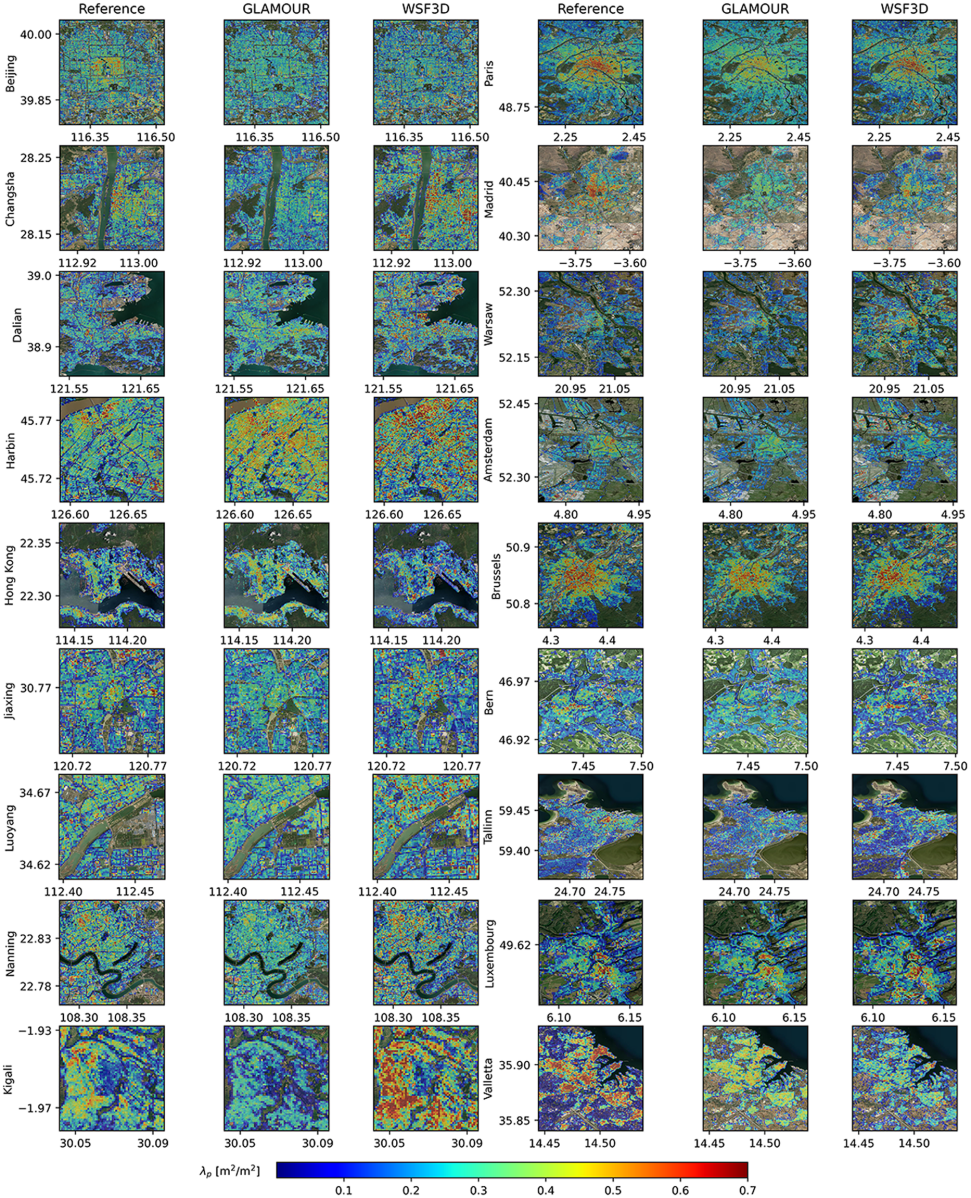
Close-up views of building footprints (*λ*_*p*_) at reference sites.

To examine the quality of the GLAMOUR dataset, we conduct validation procedures by quantifying its error against available reference datasets and comparing the corresponding performance with a recently released WSF3D dataset^12^ (https://download.geoservice.dlr.de/WSF3D/files/), which has a similar spatial coverage and a close representative year to the current work. According to the validation results in 19 globally distributed regions, the WSF3D dataset achieves average root mean square errors (RMSEs) of 6.04 m for H_avg_ and 0.1409 for *λ*_*p*_. However, the WSF3D dataset exhibits performance degradation in East Asian countries such as Korea and China^12^ where complex urban layouts and substantial variations in building morphology pose challenges to model estimation capabilities^36^. Meanwhile, during the development of SHAFTS^16^, we have validated the performance of MTDL models using a dataset primarily made up of North American cities. Building on aforementioned efforts, we further select 18 cities including 8 cities from China, 1 city from Rwanda and 9 cities from European countries considering the current availability of reference data and needs for comprehensive dataset validation. It should be highlighted that all selected cities are excluded from the stage of model development including model training and hyperparameter finetuning. Thus, this validation can offer a comprehensive assessment of the generalization ability of the MTDL model with respect to the task of global building morphology mapping. Regarding the choice of reference datasets, we select EUBUCCO (v0.1)^37^ in Europe and include target countries where more than 95% buildings have available height attributes. In China, we select the building layer from the Baidu map service (www.map.baidu.com) as the source of reference data where building height is derived from the number of floors assuming that each floor is 3.0 m^17,38^. Visual representations of the estimated building height and footprint in 18 cities can be found in Figs. 3 and 4, respectively.

### Overall performance comparison

To quantify the overall performance of the GLAMOUR dataset, we select several error metrics, including the Root Mean Square Error (RMSE), Mean Error (ME), Pearson correlation coefficient (CC), each defined as follows:

3 $${\rm{RMSE}}=\sqrt{\frac{1}{N}{\sum }_{i=1}^{N}{\left({\widehat{y}}_{i}-{y}_{i}\right)}^{2}}$$

4 $${\rm{ME}}=\frac{1}{N}{\sum }_{i=1}^{N}\left({\widehat{y}}_{i}-{y}_{i}\right)$$

5 $${\rm{CC}}=\frac{{\sum }_{i=1}^{N}\left({\widehat{y}}_{i}-\bar{\widehat{y}}\right)({y}_{i}-\bar{y})}{\sqrt{{\sum }_{i=1}^{N}{\left({\widehat{y}}_{i}-\bar{\widehat{y}}\right)}^{2}}\sqrt{{\sum }_{i=1}^{N}{({y}_{i}-\bar{y})}^{2}}}$$

where *y*, $\widehat{y}$ denote the reference values and predicted values from datasets including GLAMOUR and WSF3D. $\bar{y},\bar{\widehat{y}}$ are the mean value of *y*, $\widehat{y}$. N is the number of pixels in the mapping area.

Table 2 presents a detailed comparison of two datasets on the performance of building height and footprint estimation at each selected site. Compared to the WSF3D dataset, the GLAMOUR dataset has a better overall performance featuring a reduced magnitude and variation of RMSE for both building height and footprint estimations. Specifically, the median RMSEs achieved by the GLAMOUR dataset are 7.5 m for H_avg_ and 0.14 for *λ*_*p*_, with corresponding standard deviations of 3.9 m and 0.02. The improved performance can be benefited from the utilization of the MTDL model within the GLAMOUR dataset, which automatically learns representative features for buildng morphology mapping from various sample sites^16^, as opposed to the WSF3D dataset’s reliance on handcrafted processing workflows that might not adequately capture diverse building morphology characteristics. When examining the systematic estimation bias as indicated by the ME, the GLAMOUR dataset generally overestimates H_avg_ (such as Luxemburg in Fig. 3) and underestimates *λ*_*p*_ (such as Kigali in Fig. 4): among 18 reference sites, 72.2% show a positive ME for building height estimations and 66.7% of them have a negative ME for *λ*_*p*_ estimations. Nonetheless, the GLAMOUR dataset maintains a more stable performance with less variation in the MEs for both building height and footprint estimations. Considering the ability in capturing variation of building height distribution, the GLAMOUR dataset provides more consistent estimations compared to the WSF3D dataset, with a median CC of 0.54, suggesting a moderate statistical correlation between the predicted and reference maps. In regard to building footprint, two datasets achieve comparable results with a median CC of 0.52, though the GLAMOUR dataset shows a slightly larger performance variation.

**Table 2:** Validation results for the building footprint (*λ*_*p*_), building height (*H*_avg_) in 18 reference sites where the suffix of “G” and “W” denote the GLAMOUR and WSF3D dataset, respectively.

Reference Site	*H*_avg_ [m]						*λ*_*p*_ [m^2^/m^2^]					
	RMSE_G_	RMSE_W_	ME_G_	ME_W_	CC_G_	CC_W_	RMSE_G_	RMSE_W_	ME_G_	ME_W_	CC_G_	CC_W_
Beijing (CHN)	10.6	15.8	1.3	−3	0.7	0.42	0.15	0.18	−0.02	0.01	0.48	0.36
Changsha (CHN)	14.3	20.4	3.4	−2.5	0.54	0.24	0.13	0.2	−0.02	0.08	0.56	0.37
Dalian (CHN)	11.7	16.9	1	−2.3	0.44	0.2	0.14	0.2	0.03	0.09	0.42	0.36
Harbin (CHN)	11	15.7	−0.9	−2.2	0.37	0.27	0.18	0.2	0.08	0.08	0.34	0.44
Hong Kong (CHN)	20.5	42.9	2.4	20.8	0.5	0.5	0.15	0.16	0.04	0	0.49	0.46
Jiaxing (CHN)	8.7	11.5	1.3	−2.5	0.41	0.1	0.16	0.16	−0.01	0	0.26	0.43
Luoyang (CHN)	12.7	17.9	−4.4	−6.5	0.64	0.26	0.13	0.17	0.01	0.06	0.46	0.47
Nanning (CHN)	11.9	17.4	3	−0.9	0.56	0.3	0.14	0.19	-0.01	0.04	0.48	0.41
Kigali (RWD)	4.7	3.9	3.6	2.1	0.18	0.42	0.16	0.21	−0.1	0.14	0.48	0.68
Paris (FRA)	5.3	7.6	1	−0.7	0.68	0.62	0.13	0.14	−0.01	0	0.63	0.67
Madrid (ESP)	7.4	8.3	3.5	1.7	0.6	0.58	0.18	0.2	−0.06	−0.02	0.59	0.52
Warsaw (POL)	6	8.3	−0.4	−2	0.66	0.6	0.12	0.17	0	0.08	0.53	0.53
Amsterdam (NLD)	5.7	7.4	0.8	−1.1	0.58	0.6	0.18	0.18	−0.06	−0.01	0.51	0.55
Brussels (BEL)	6.8	8.2	4.1	2.3	0.35	0.34	0.13	0.16	−0.04	−0.02	0.74	0.63
Bern (CHE)	5.6	6.2	3.2	−1.3	0.29	0.3	0.1	0.13	−0.02	−0.04	0.57	0.57
Tallinn (EST)	7	8.8	−2.3	−4.8	0.54	0.55	0.14	0.16	−0.05	−0.01	0.56	0.51
Luxembourg (LUX)	7	5.7	5.2	1.5	0.63	0.61	0.12	0.15	0	0.02	0.67	0.6
Valletta (MLT)	7.6	9.6	−4.1	−6.9	0.51	0.5	0.18	0.22	−0.09	−0.12	0.67	0.54
**Median**	**7.5**	9.2	**1.3**	−1.6	**0.54**	0.42	**0.14**	0.18	−0.02	**0**	0.52	0.52
**Standarddeviation**	3.9	8.7	2.7	5.8	0.14	0.16	0.02	0.03	0.04	0.06	0.12	0.10

While the GLAMOUR dataset marks an advancement over existing datasets, there remains several undesirable cases with considerably worse performance in both datasets such as the mappings of building height in Hong Kong and footprint in Valletta. For the case of Hong Kong (as illustrated in Fig. 3), both datasets overestimate H_avg_, especially for the WSF3D dataset with a dramatically higher ME of 20.8 m, possibly due to the over-correction caused by empirical adjustments designed for high-rise buildings during its generation^12^. Furthermore, Hong Kong is characterized by densely packed high-rise buildings over a hilly topography^39^, posing a significant challenge for accurate building morphology mapping with medium-resolution satellite data such as Sentinel-1/2 imagery. Although the GLAMOUR dataset reduces nearly half of the RMSE compared to the WSF3D dataset, it still requires further improvement, particularly in the central northern area (known as Kowloon Tong) featuring relatively lower buildings. For the case of Valletta (Fig. 4), both datasets tend to underestimate *λ*_*p*_ where the GLAMOUR dataset achieves a slightly better result with a RMSE of 0.18. Closer examination of high-resolution satellite images from Valletta, especially in zones with *λ*_*p*_ greater than 0.7, reveals a pattern of mid-rise buildings with minimal spacings, forming large building bulks often individually labeled as combined buildings in EUBUCCO (v0.1). Such configuration in building morphology may hinder the WSF3D dataset from detecting existing building structures using focal windows with kernel sizes up to 60 m around the center pixel^12^. The MTDL model adopts a larger input patch size of 200 m to produce the GLAMOUR dataset and thus can benefit from a wider receptive field to improve the performance of 3D morphology estimations in building combinations.

### Stratified performance comparison

**Fig. 5:**
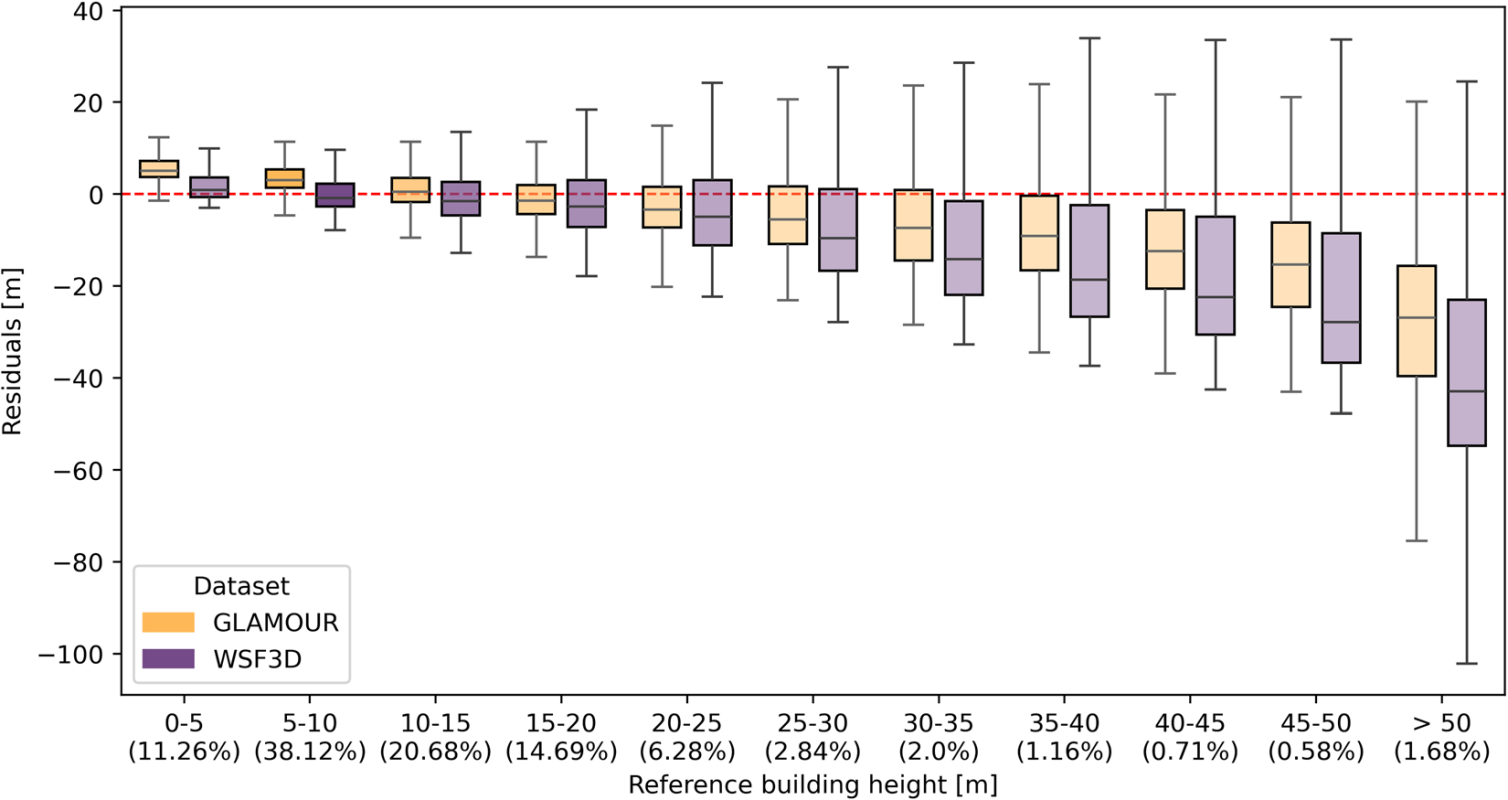
Mapping residuals of the GLAMOUR and WSF3D datasets across building height values aggregated with 5 m intervals. The percentages of validation samples are labelled at the x-axis and indicated by the transparency of boxes where darker boxes denote more samples.

**Fig. 6:**
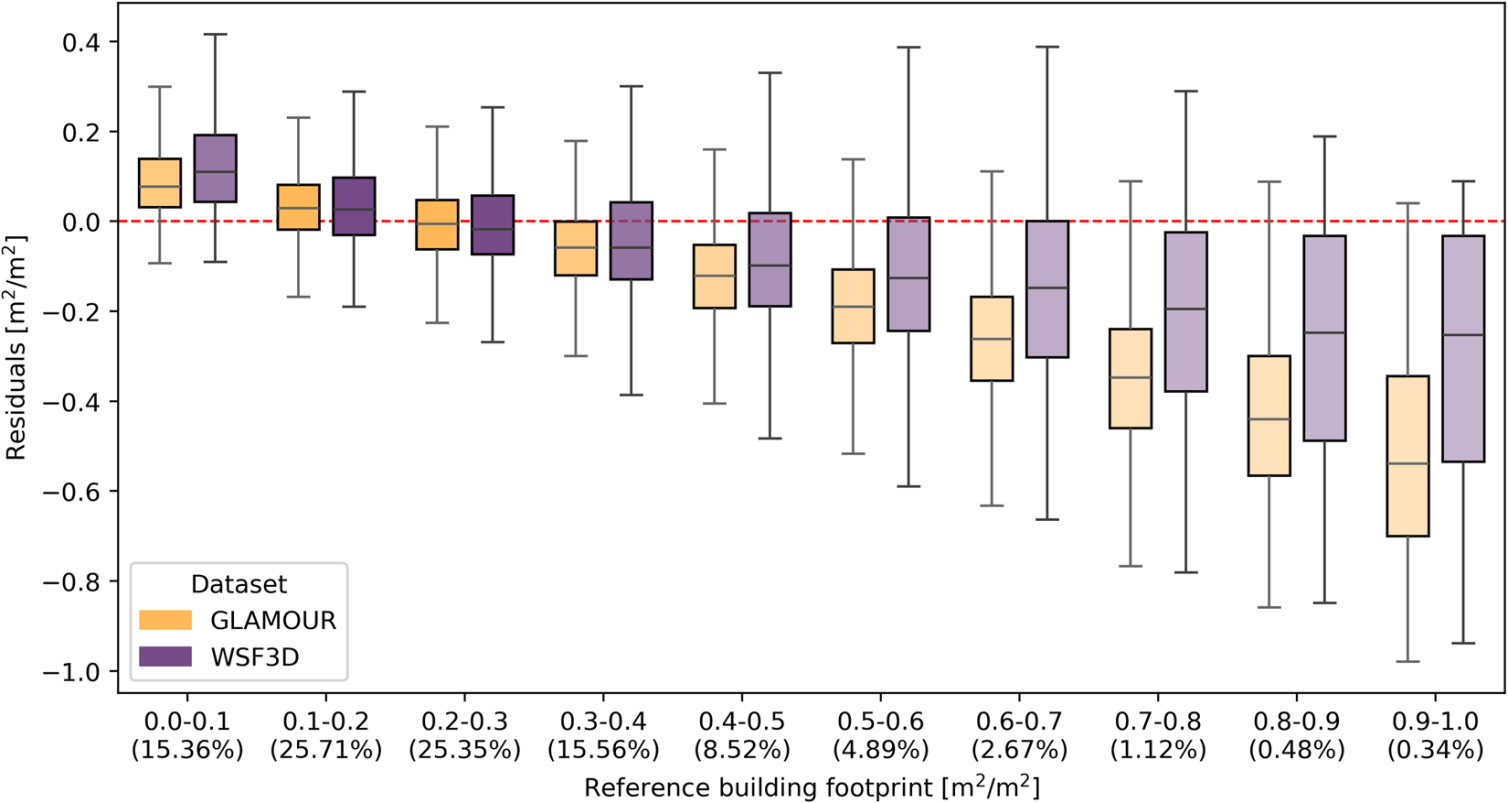
Mapping residuals of the GLAMOUR and WSF3D dataset across building footprint values aggregated with 0.1 intervals. The percentages of validation samples are labelled at the x-axis and indicated by the transparency of boxes where darker boxes denote more samples.

To thoroughly investigate the performance of the GLAMOUR dataset across various target intervals, we further perform the stratified evaluation by aggregating samples according to corresponding building height and footprint values, using bins of 5 m and 0.1, respectively. The distributions of mapping residuals, calculated as the difference between predicted and reference data, are illustrated in Fig. 5 and Fig. 6.

For building height predictions, the GLAMOUR dataset delivers better performance over the WSF3D dataset in most target intervals, demonstrating with consistently smaller magnitude and variation of residuals. Specifically, for buildings exceeding 30 m, the GLAMOUR dataset exhibits its superiority and achieves significantly smaller median residuals ranging from -7.4 m and -26.9 m, which reduce ~ 37.4% -51.1% residuals of the WSF3D dataset within the same intervals. However, we can also notice that residuals increase in intervals such as 0-5 m and 5-10 m with median values of 5.0 m and 3.0 m, respectively, indicating an overestimation tendency for the height of lower buildings with 2-3 floors in the GLAMOUR dataset.

For building footprint predictions, when compared with the WSF3D dataset, the GLAMOUR dataset exhibits comparable magnitude but reduced variation of residuals in the intervals ranging from 0.1 to 0.4, which encompasses 81.98% validation samples. This indicates a more stable performance by the GLAMOUR dataset on building footprint estimations in sparsely or moderately built-up areas. However, for the remaining proportion of samples with *λ*_*p*_ greater than 0.5, the GLAMOUR dataset shows a considerable underestimation with a median residual ranging from -0.13 to -0.25. The degradation of performance in these intervals corresponding to densely built-up areas can be attributed to different spatial resolutions of input data used by the two datasets: the WSF3D dataset combines the information from the 3 m SAR amplitude and 12 m TanDEM-X DEM to delineate the building coverage at a 12 m resolution and then generates the final 90 m dataset by zonal aggregation^12^; while the GLAMOUR dataset focuses on the publicly accessible 10 m Sentinel-1/2 images and 30 m global DEM and utilizes the MTDL model to estimate the building footprint from relatively coarser images. Thus, the WSF3D dataset can benefit from additional details originating from images with higher resolution^8^ and enhance its detection abilities of vertical structures in densely built-up areas. However, given its reliance on empirically determined rules based on backscattering characteristics reflected in the SAR amplitude images, it would face the difficulty in distinguishing building roofs from surrounding environments with similar backscattering properties (such as Kigali in Fig. 4)^12^ while the GLAMOUR dataset exhibits its potential in alleviating this issue by leveraging multi-source information from optical and radar images accompanied local elevation features and thus can achieve improved performance in certain regions with mixed building patterns. Although samples with *λ*_*p*_ greater than 0.5 only occupy a relatively small fraction of the validation dataset (around 9.5%), it still requires further improvement to address this underestimation issue, partly due to the constraints of resolution in publicly available imagery.

## Usage Notes

The GLAMOUR dataset is provided in the GeoTiff format which can be easily read, analyzed and visualized with open-source GIS softwares (e.g. QGIS) as well as Python packages (e.g. GDAL and rasterio). We provide five Python-based functions in the example module of SHAFTS (https://github.com/LllC-mmd/SHAFTS/blob/main/example/glamour.py) to facilitate working with the GLAMOUR dataset:

- get_glamour_by_extent: retrieves a subset of building morphology files within a specific geospatial extent from the GLAMOUR dataset.
- vis_glamour_by_extent_2d: visualizes the building morphology defined in the GLAMOUR dataset with close-up 2D maps (similar to Figs. 3 and 4).
- vis_glamour_by_extent_3d: visualizes the building height defined in the GLAMOUR dataset with interactive web-based geospatial maps (similar to Fig. 2).
- ana_glamour_joint_distribution: derives the joint distribution of building height and footprint within a specific geospatial extent based on the GLAMOUR dataset.
- ana_glamour_add_height_attribute: attach the average building height of the GLAMOUR dataset to the attribute table of a given building vector layer.

**Fig. 7:**
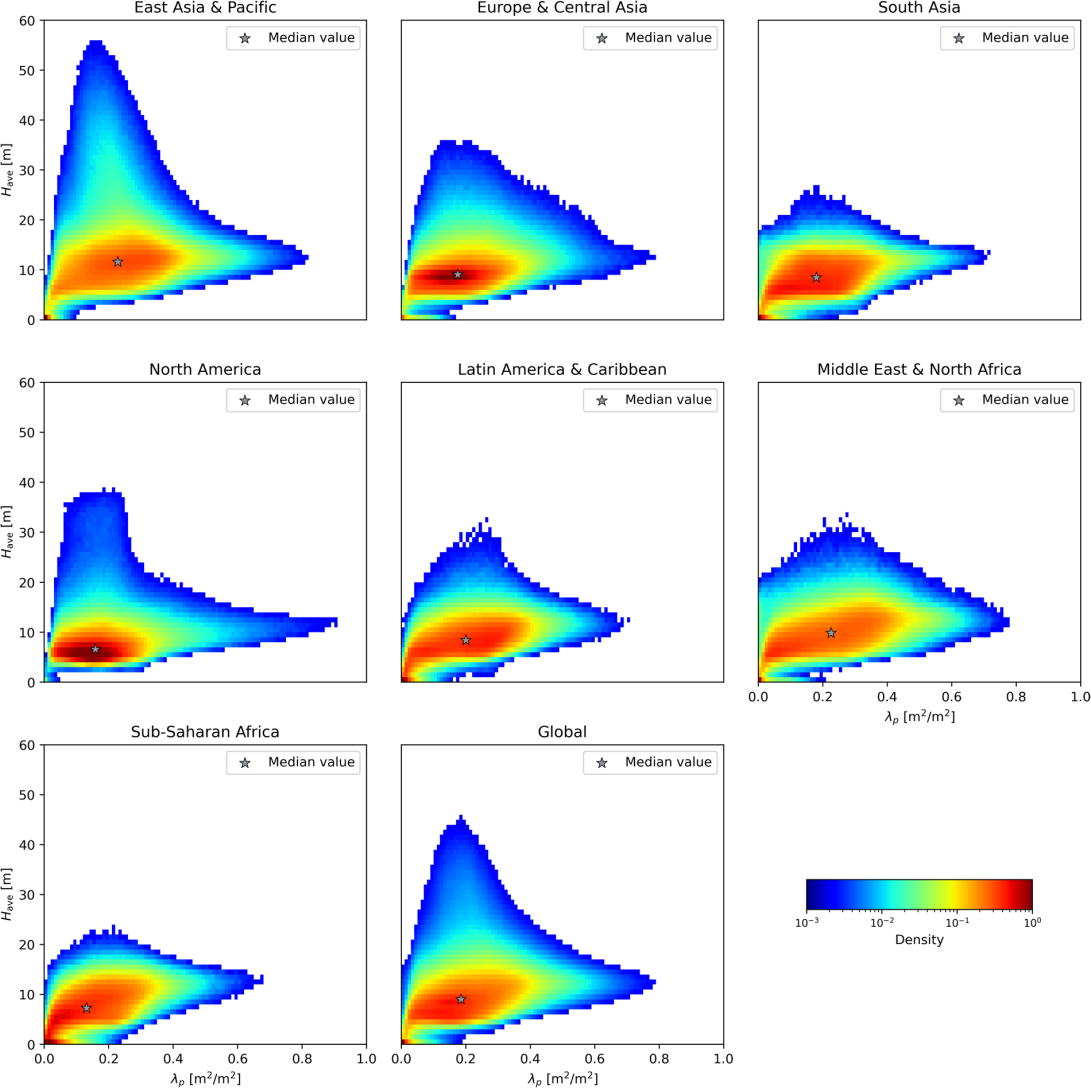
Joint probability density distribution of building footprint (*λ*_*p*_) and height (H_avg_) in urban centers across global regions (data points with density below 0.002 excluded). The region of urban centers are derived from the world bank country classification (https://datahelpdesk.worldbank.org/knowledgebase/articles/906519-world-bank-country-and-lending-groups).

Figure 7 exhibits the distribution of 3D building morphology in 13189 urban centers around the world. The results of quantitative analysis show that when mapped at a resolution of 0.0009^°^, the median building height is 9.0 m and the median building footprint is 0.19, with standard deviations of 5.9 m and 0.12, respectively. This indicates that the majority of urban centers are still dominated by open low-rise buildings^40^. Among seven regions displayed in Fig. 7, the East Asia and Pacific region has the highest median values for both building height and footprint at 11.6 m and 0.23, respectively. Conversely, the North America region has the lowest median building height of 6.6 m and the Sub-Saharan Africa has the lowest median building footprint of 0.13, which exhibits regional variation in building morphological patterns influenced by local urbanization stages and socioeconomic factors. From a visaul analysis of density plots, it appears that the East Asia and Pacific region is characterized by vertical expansion, as evidenced by wide spreading of building height with varying building footprint. In contrast, the North America region predominantly features low-density sprawlings of urbanized areas scattered with high buildings.

Beyond basic analysis of building morphology, the GLAMOUR dataset offers further support for the derivation of morphometric parameters for urban hydroclimate simulation. To maximize its effectiveness in such modeling uses, it is recommended to integrate the GLAMOUR dataset with other vectorized building footprint datasets with global coverage such as Global ML Building Footprints (https://github.com/microsoft/GlobalMLBuildingFootprints), along with existing urban climate service tools such as the Urban Multi-scale Environmental Predictor (UMEP)^41^ (https://umep-docs.readthedocs.io/en/latest/). For instance, we can match individual buildings with gridded values of average building height in the GLAMOUR dataset using the provided ana_glamour_add_height_attribute function. Once a vectorized building footprint layer is associated with corresponding height attributes, it can be further processed within the UMEP framework, which includes the DSM generator for generating the DSM consisting of ground and buildings, and the morphometric calculator for deriving desired morphometric parameters such as roughness length and zero-plane displacement height prepared for urban hydroclimate simulation.

## Data Availability

The generation of the GLAMOUR dataset is based on SHAFTS, Google Earth Engine, Google Cloud Storage and their Python interfaces. The snapshot of the source code used in this study has been archived on Zenodo (10.5281/zenodo.10608714). And the up-to-date streamlined workflow for large-scale building morphology mapping can be accessed via the GBuildingMap function in SHAFTS (https://github.com/LllC-mmd/SHAFTS).
